# Bioimaging: Evolution, Significance, and Deficit

**DOI:** 10.7759/cureus.28923

**Published:** 2022-09-08

**Authors:** Harsh S Lahoti, Sangita D Jogdand

**Affiliations:** 1 Medicine, Jawaharlal Nehru Medical College, Datta Meghe Institute of Medical Sciences, Wardha, IND; 2 Pharmacology, Jawaharlal Nehru Medical College, Datta Meghe Institute of Medical Sciences, Wardha, IND

**Keywords:** metabolism, mechanical motion, electric and magnetic fields, anatomical structure, bioimaging

## Abstract

Bioimaging is a digital technology-based medical advancement which is still relatively new. It has to do with real-time visualization of biological processes. This innovative imaging technology combines anatomical structure with functional data such as electric and magnetic fields, motion which is mechanical, and metabolism to provide information on anatomical structure. It's a non-invasive procedure that gives you a bird's-eye view of the human body, with more depth and detail as you go. As a result, bioimaging is a strong tool for seeing the interior functioning of the organism and its disorders. Examples of bioimaging in the medical industry include X-ray and ultrasound pictures, MRI, 3D and 4D body images utilizing Computed Tomography (CT) scans, DEXA scans which is useful for assessing bone density in osteoporosis, and so on. Maximum-resolution, two-positive charge fluorescent excitation microscopy, fluorescence redistribution after photobleaching, and fluorescence resonance energy transfer are some of the recent advancements in biological imaging. It provides us a cellular-level means of obtaining photographs of the entire body, anatomical locations, organs, tissues, and biological indicators. It may be used to aid illness management and therapy, as well as to detect, diagnose, and characterize the problems in clinical settings.

## Introduction and background

Bioimaging is a term that refers to a procedure in which there is no involvement of tools that can invade the skin or physically enter the body, and it allows scientists to view biological functions in real time. The purpose of bioimaging is to cause as minimal disruption to live processes as possible. It is also widely used to get data on the three-dimensional structure of the viewed item without requiring physical interaction [1]. In a broader sense, bioimaging refers to technologies for viewing biological substances that have been fixed for monitoring. In the fundamental and medical sciences, bioimaging can be used to examine typical anatomy and physiology and gather research data. Due to the multifaceted nature of bioimaging research, interdisciplinary teams with expertise in electrical engineering, mechanical engineering, biomedical engineering, and other fields are required [2].

Multi-modal (such as combined ultrasound and light imaging) and multi-scale imaging is frequently needed for complex bioimaging applications (e.g., molecular to cellular to organ). Imaging makes it possible to understand intricate structures and dynamic interacting processes deep within the body. Many imaging techniques make use of the entire energy spectrum. Examples of clinical modalities are ultrasound, CT using X-rays, optical coherence tomography (OCT), and MRI [3]. Research methods include, among others, electron microscopy, mass spectrometry imaging, fluorescence tomography, biochemical luminescence, various forms of OCT, and optoacoustic imaging. Light microscopy methods include confocal, multi-photon, total internal reflection, and super-resolution fluorescence microscopy [4]

## Review

The evolution of medical imaging has a long history that dates back to the 1890s. It grew in popularity in the 1980s and has been extensively researched in recent years due to technical improvements [5]. The same fundamental concept underlies all imaging techniques. The body or region to be diagnosed is traversed by a wave beam that transmits or reflects radiation. A detector arrests this radiation and handles it to create a depiction pattern. The wave format varies in various procedures [6]. While CT employs X-rays, MRI and single-photon emission CT (SPECT) use radio frequency waves and gamma rays [5]. The area of biomedical imaging has advanced over the past 100 years, starting with Roentgen's initial discovery of the X-ray and ending with a new imaging approach with MRI, CT, and PET. A brief overview of different bioimaging techniques is discussed. Some of the technologies under investigation at the moment are magnetic resonance spectroscopy (MRS), functional MRI, diffusion-weighted MRI, and molecular imaging. Examples of molecular imaging methods include PET, SPECT, and optical imaging [7].

About 400 years back, multiple-lens microscopes were invented and employed in medical research. Employing computational methods for picture analysis, researchers were able to utilize their potential fully after the invention of digital photography. As digital technology advances, bioimaging is anticipated to become cheaper, quicker, and the backbone of medical research. Bioimaging may be broadly differentiated into four categories; they are molecular bioimaging, biomedical imaging, bioimaging in drug discovery, and computational bioimaging [8].

X-ray and ultrasound pictures

**Figure 1:**
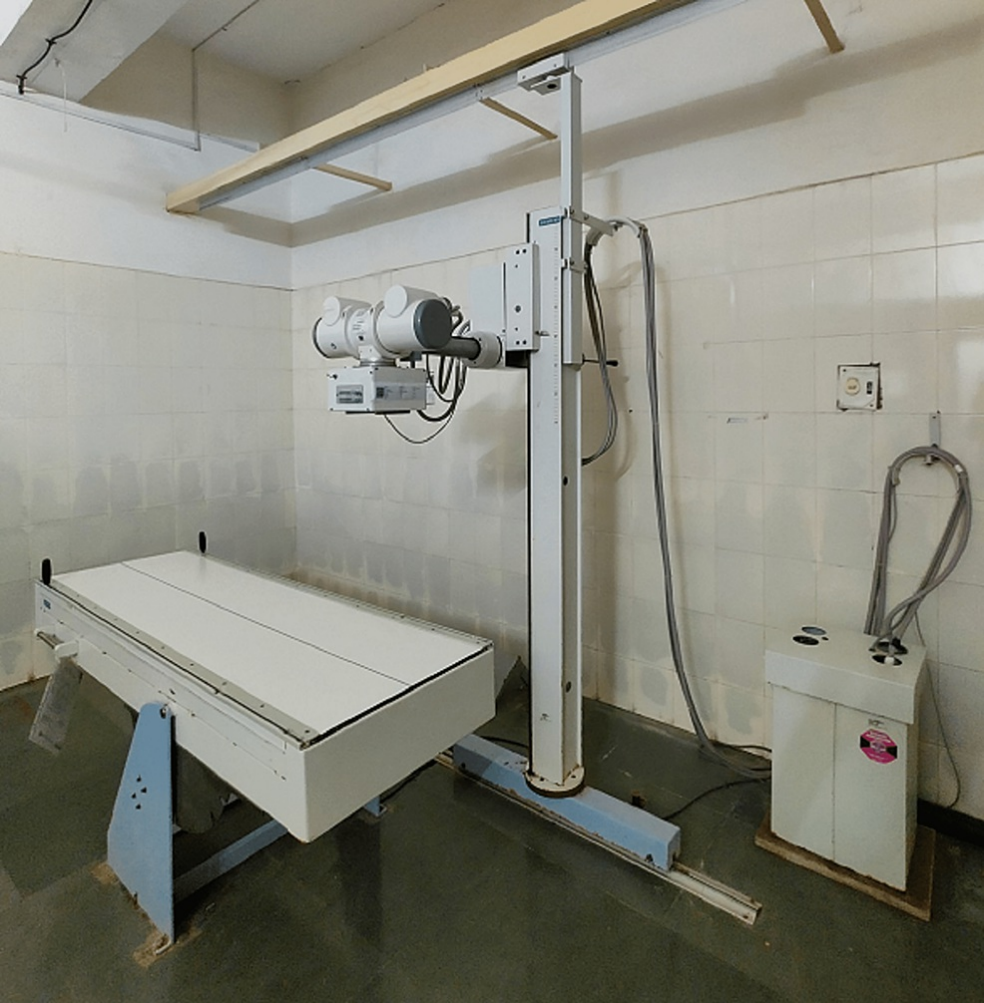
X-ray machine Image credit: Harsh Lahoti

X-rays have been the most popular, frequently quickest, and inexpensive therapeutic imaging method since Wilhelm Conrad Röntgen discovered them in 1895. The medical X-ray imaging equipment portfolio has expanded into a wide range of specialized equipment for many purposes, starting with early radiographic systems that employed X-ray films as detectors. Although X-ray sources release a broad variety of X-ray image energies and X-ray image collisions within the human body vary for each energy and substance, most X-ray imaging done today is in black and white [9]. Figure 1 shows a modern view of an X-ray machine currently used in practice in institutes all over the world for the purpose of diagnosis.

X-ray medical imaging has two major categories: structural images that reveal anatomical structures and functional ideas that measure changes in biological functions such as metabolism, blood flow, local chemical composition, and biochemical processes. They're commonly utilized to image the structure of bone, metal implants, and soft tissue cavities [10]. Radiography very often plays a crucial part in evaluating the several bony structures of the body; however, it is beyond the scope of this article to adequately describe the complete spectrum of uses of conventional radiographs. It is also possible to evaluate the lungs, and contrast can aid in examining soft tissue organs throughout the body, such as the uterus and the gastrointestinal system, as in the case of hysterosalpingography [11]. Stereotactic breast biopsies, intra-articular steroid injections, catheter angiography, and other operations can all be performed with the help of radiography. Numerous diseases, including fractures, different forms of pneumonia, cancers, and congenital anatomic anomalies can be evaluated with radiography. Some X-ray developments for clinical uses are mammography, diagnosis of arthritis, and diagnosis of lung disease [12].

MRI

**Figure 2:**
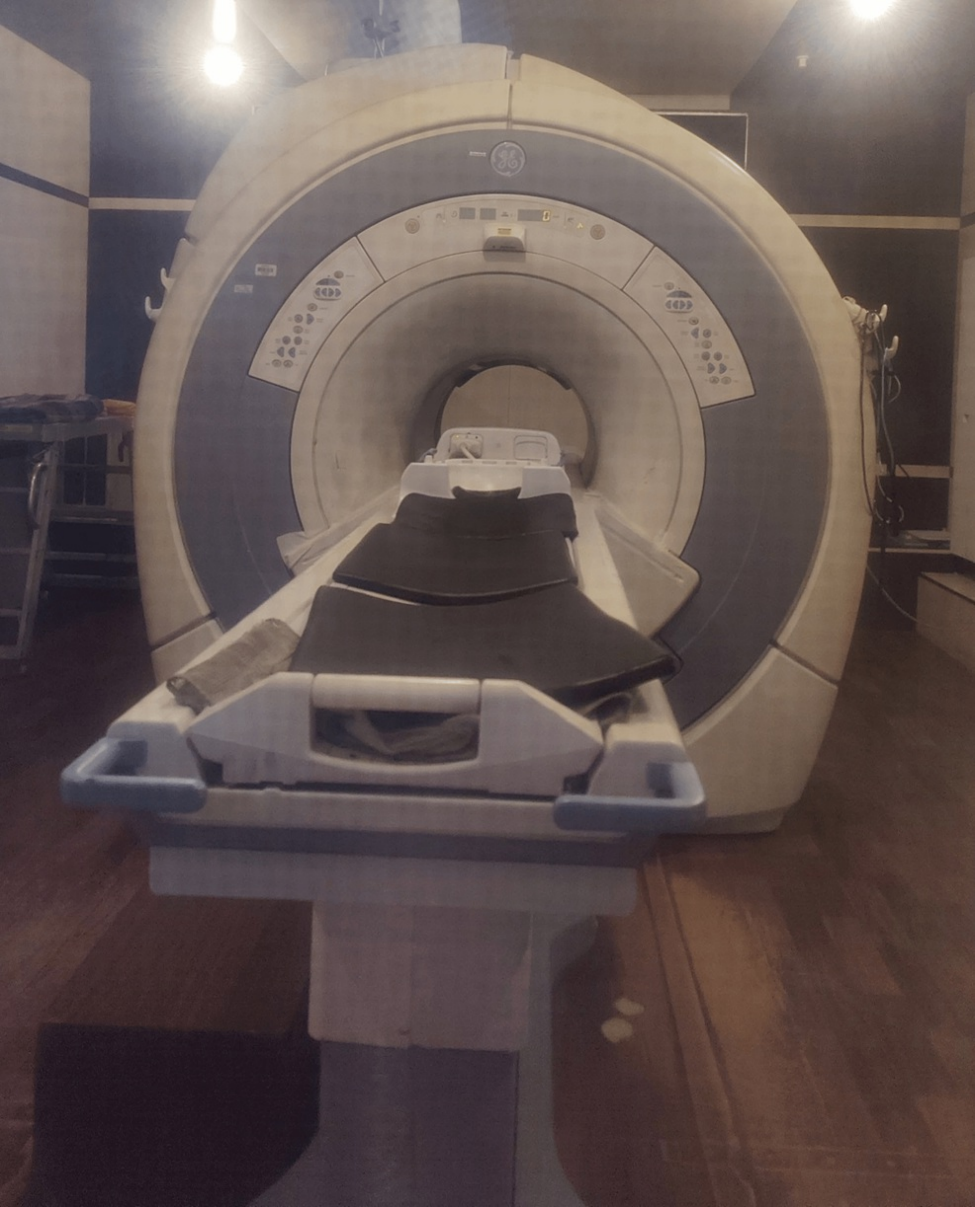
MRI machine Image credit: Harsh Lahoti

As it generates high-resolution pictures and greater soft tissue dissimilarity, MRI is preferably utilized for detection instead of CT, ultrasound, and X-ray. MRI can also scan any 2D sections or 3D volume of the body in a sense according to the need of the patient to move between scans [13]. Recently MRI with greater than three Tesla are used for examination of temporomandibular joint pathologies like fine perforations of articular disk and fibrinous adhesions of temporomandibular joint (TMJ). As a result, an MRI may be used as a guide tool through the body during medical operations. MRI-guided operations can streamline the whole process and benefit doctors and patients by allowing diagnosis, medication, and assessment after procedure to all be completed in only clinical workflow [13]. Figure 2 shows the view of currently used MRI machine in the majority of health sector areas for the diagnosis of disease.

It does not employ radiation, unlike CT. MRI's diagnostic capabilities are constantly being improved. MRI with greater than two Tesla is developed for more precision. MRI has replaced CT in various sectors and has multiple uses. With a few exceptions, MRI is generally used for optional tests, and it is growing more common as new technologies like diffusion and perfusion become available. For work-up and follow-up, the usage of MRI in cancer imaging is increasing continuously. MRI is highly used for non-malignant lesions, imaging vertebral anomalies, meninges of the brain, namely pia mater, dura mater, and arachnoid membranes covering the brain, intracranial tumours, and fine soft tissue details, due to its great soft-tissue dissimilarity potentiality [14].

Merits of MRI

An MRI scanner may be used to grasp snapshots of different sections of the body (e.g., skull, abdomen, joints, lower limbs, etc.) in various directions of imaging. The MRI scans help the clinician in easy prognosis of vivid illnesses [15].

Demerits of MRI

Radiation revelation during an MRI operation is not a concern since ionizing radiations are not used. As MRI uses a very powerful magnet, it should not be done on those who have heart and other pacemakers inserted, clips for intracranial aneurysms, implanted cochlea, a few artificial limbs, surgically implanted infusion pumps, neurostimulators, bone-growth promoters, any other iron-based metal implants, or a few different types of intrauterine devices. MRI is also contraindicated in patients having surgical clips, screws, metallic plates and sutures, wire mesh, and metallic pins in their bodies. Metallic objects which are used internally, like shrapnel or bullet, are also denied. You should let your clinician know whether you are carrying a baby in the womb or think you might be. MRI also has demerits of claustrophobia (fear of closed place) and noise created during imaging. MRI is generally not proposed for individuals during gestation because of the risk of the elevation in the amniotic fluid's temperature [16].

CT Scan

**Figure 3:**
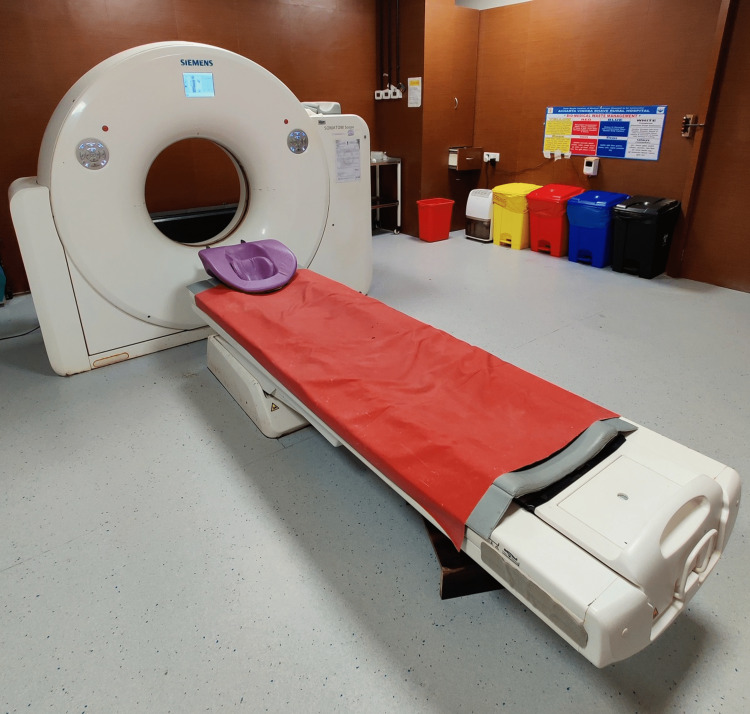
CT machine Image credit: Harsh Lahoti

CT has changed diagnostic decision-making since its inception in the 1970s. It has improved surgery, cancer detection, and therapy, more preferred treatment after an accident and significant injury, stroke therapeutics, and cardiac care [17]. To create cross-sectional pictures, or "slices," of the patient's body, a small X-ray beam is focused on the patient and rapidly rotates around the body. The CT scan paved the way for more effective treatment of deadly conditions like cancer, strokes, heart, problems, orthodontics, and accident injuries. Due to the COVID-19 outbreak, CT was recently utilized to diagnose patients who had been diagnosed for lung infection viral pneumonia caused by COVID-19 and it was shown to be very sensitive [18]. Figure 3 shows the view of currently used CT machine in various places for detection of fatal conditions.

A variety of tiny, distinct bodily components are measured using CT to determine their radiographic densities. In a typical tomography slice, each of these components is represented by a pixel, which is then used to display the elements of a two-dimensional image. In order for the radiographic density of attentiveness to appear between dark and light in the visual picture, the operator allots a range of grey shades between black and white to a specific range of densities [19].

For most of the brain investigations, MRI has replaced CT. However, it is still the preferred imaging method for acute cranial trauma. For the viscera in the abdomen, CT is often preferable to MRI. The evaluation of solid organs is more debatable. Modern CT and MRI are competitive for the liver, spleen, kidneys and perhaps the pancreas. For the pelvic organs, MRI is better. The test will depend on the area's competence, equipment accessibility, cost, and radiation exposure [20].

Photon-counting CT is an emerging technology with the potential to change clinical CT dramatically. Photon-counting CT uses new energy-resolving X-ray detectors with mechanisms that differ substantially from conventional energy-integrating sensors. Photon-counting CT detectors count the number of incoming photons and measure photon energy. This technique results in a higher contrast-to-noise ratio, improved spatial resolution, and optimized spectral imaging. Photon-counting CT can reduce radiation exposure, reconstruct images at a higher resolution, correct beam-hardening artefacts, maximize the use of contrast agents, and create opportunities for quantitative imaging relative to current CT technology [21].

Advanced bioimaging techniques

In the past few years, there has been much more advancement in bioimaging techniques which help a physician in quicker and more efficient diagnosis.

Super-resolution

From the observed lateral resolution pictures, the super-resolution approach rebuilds a very higher resolution image [22]. Because super-resolution has been around for almost thirty years, both multi-frame and single-frame super-resolution have essential uses in our day-to-day lives [23]. Super-resolution contributes to the resolution of this problem by generating high-resolution MRI from otherwise low-quality MRI images. Super-resolution is utilized to distinguish repetition from different sources that are closer than the usual diffraction limit. A higher capture frequency was combined with this search, which improved resolution. The ability to discern between two objects, in this case, vessels, beyond the traditional limit is super-resolution. The application will eventually decide whether or not any of the customary limits are applicable. Most people believe the lower resolution limit is represented by the diffraction barrier at half wavelength. Utilizing the 1.22 (focal length/aperture) Rayleigh resolution criteria is an additional choice. Even if the latter definition is typically more flexible, achieving a 150-micron resolution at 15 centimetres depth with a 5 centimetres aperture 5-Mega Hertz transducer is still an exciting exploitation for therapeutic implementation, mainly for more enormous organs. In both circumstances, the specific limitation has to be described in the publication precisely so that research may be compared more easily [24].

Advantages of contemporary super-resolution microscopy: The research of sub-cellular architecture and dynamics at the nanoscale is made possible by super-resolution microscopy. Both the sample's surface and its interior, which extends up to 100 m deep, are readily visible to researchers. Thanks to improved temporal resolutions, time-lapse imaging allows researchers to collect precise three-dimensional super-resolution picture data. Some super-resolution microscopy techniques combine intrinsic optical sectioning with quick data capture and dual-colour super-resolution to deliver high-quality pictures quickly for further actions [25].

Disadvantages of contemporary super-resolution microscopy: At higher resolutions, spherical aberration and vibration are even more problematic. Additionally, because of their high excitation intensity or lengthy exposure durations, certain living samples are more negatively impacted by super-resolution imaging than others. Another issue with many super-resolution systems is their lack of adaptability; if an experimental procedure changes in the midst of an application, many hardware-based super-resolution systems like spatial resolution or pixel density and charge-coupled device are difficult to adjust [26].

Fluorescence Recovery/Redistribution After Photobleaching (FRAP)

Since it was initially brought into cell biology research, the phenomenon of FRAP has gotten a lot of interest. The approach was created in the 1970s, and its biological applicability was limited to the mobility of fluorescently labelled cell membrane components. In the 1980s, the introduction of confocal scanning microscopy made it possible to study the behaviour of molecules inside cells without specialized equipment. However, FRAP has not gained widespread acceptance till date, owing to the time and effort necessary to extract, label and, inject proteins and other chemicals into cells [27].

In contrast to FRAP investigations, bleaching is carried out repeatedly at the same region of the material in fluorescence loss in photobleaching (FLIP), which prevents fluorescence recovery. The area of interest has not yet been bleached [28]. Cell biology provides instances of this in transporting proteins and lipids in the plasma membrane, cytoplasm, and nucleus. The qualities and usefulness of the finished product in commercial applications, including medicines, food, textiles, sanitary goods, and cosmetics, are considerably enhanced by the diffusion of solute and solvent molecules [29,30]. Since each of these systems is different, precise local measurements of mass transport processes are required to comprehend the characteristics of soft biomaterials. FRAP uses fluorescence microscopy to measure regional molecular mobility on a micrometre scale [31].

Fluorescence Resonance Energy Transfer (FRET)

Fluorescence resonance irradiative energy is transmitted from an excited molecular fluorophore (donor) to another fluorophore (acceptor) via long-range intermolecular interactions linking between dipoles in the energy transfer process. FRET can be a trustworthy technique for finding out molecular closeness at angstrom distances (10-100) if the donor and acceptor are situated between the donor and acceptor's Forster radius, which is typically 3-6 nm and is the interval at which the donor's excitation energy is split in half and passed to the acceptor. FRET offers a sensitive approach for analyzing a range of biological activities that affect molecule closeness since its effectiveness depends on the inverse sixth power of intermolecular separation [32].

In the fluorescence resonance, the energy transfer process and excited donor fluorophore can non-radiatively transfer its excitation energy to neighbouring acceptor chromospheres through long-distance dipole-dipole interactions [33]. According to the energy transfer theory, an activated fluorophore acts as an oscillating dipole that may exchange power with another dipole with a resonance frequency close to its own. Similar resonance energy transmission occurs in coupled oscillators that oscillate at the same frequency, such as a pair of tuning forks. The release and reabsorption of photons required for radiative energy transfer are controlled by the specimen's geometrical and optical properties and by the container's structure and wavefront paths [34].

Over the last two decades, emerging biological imaging technologies have generated tremendous biological discoveries, many of which directly rely on computational methodologies. The development of image informatics solutions, from capture to storage, analysis to mining, and visualization to distribution is critical to the future of biological imaging innovation. Continued development of computational methods for bioimaging will not only pave the way for new imaging technologies but also enable biological breakthroughs that would not otherwise be possible. For the thousands of scientists who depend on bioimaging, investing in the creation and upkeep of essential software programs and the connections between them will pay off handsomely [35]. Despite the benefits of bioimaging, using microscopes has several disadvantages. Aside from the cost of acquiring, storing, and maintaining equipment, sample preparation is often required. For example, in field emission scanning electron microscope (FESEM), the sample has to first be dried and disseminated with gold particles, which is time-consuming and tedious. Furthermore, methods like FESEM's high resolution might sometimes be a double-edged sword. FESEM for example was particularly useful in the previous work for examining the structure and interphase between the bacteria and the cell. It was difficult to hunt for small quantities of bacteria when the researchers used less inoculants to investigate the cells for 23 hours to determine if they survived. Complementary methods, such as fluorescence microscopy, are frequently required in situations like these to acquire an overview of the material [36].

Application of artificial intelligence (AI) in radiology

Radiology practice will undergo a significant transition as a result of deep learning and AI technologies' quick development and integration into routine clinical imaging [37]. AI is increasingly playing a significant role in various health care applications, such as drug development, remote patient monitoring, medical imaging and diagnostics, risk management, wearable technology, virtual assistants, and hospital administration. It is also anticipated that the application of AI would be advantageous in many fields involving massive data, such as processing data from DNA and RNA sequencing. Radiology, pathology, dermatology and ophthalmology are just a few medical specialities that rely on imaging data and they have already started to gain from using AI techniques. Examples of AI clinical applications in radiology are thoracic imaging, abdominal and pelvic imaging, head and neck imaging, pathologies around teeth, colonoscopy, brain imaging, mammography, and many more [38]. The boon of AI in radiology is that the radiologist may focus on challenging situations requiring specialized attention by letting AI handle a sufficient portion of the image diagnostic workload. The weight of image diagnosis can be sufficiently assumed by AI, freeing the radiologist to concentrate on the complicated situations that need their specialized attention. AI helps the teleradiologist and also decreases burnout. It is used to diagnose and assess patients over great distances, reducing the time it takes to evacuate emergency patients from rural and isolated locations. One of the demerits of AI is it lacks human empathy [39].

## Conclusions

Bioimaging gives clinicians a chief tool for checking patients' reactions to therapy. It promises illness detection in therapy in a non-invasive and safe manner. Bioimaging is a very important innovative imaging technology that has a lot of significance in today's world. The designing policies for imaging inquest are required for accurate imaging to access effective cancer management both in vitro and in vivo. It is of utmost importance in medical sciences as it has advanced the process of diagnosis of various diseases. It has helped to prevent a large number of illnesses and many more complications. It helps clinicians in early diagnosis to prevent future consequences. In this article, we have discussed various bioimaging techniques and their advancement. We have also discussed the merits and demerits of bioimaging techniques. The introduction of bioimaging in medical sciences has proved to be an asset to the world. 
